# Relationship between BMI and emotion-handling capacity in an adult Finnish population: The Northern Finland Birth Cohort 1966

**DOI:** 10.1371/journal.pone.0203660

**Published:** 2018-09-26

**Authors:** Nurul Hanis Ramzi, Andrianos M. Yiorkas, Sylvain Sebert, Sirkka Keinänen-Kiukaanniemi, Leena Ala-Mursula, Rauli Svento, Jari Jokelainen, Juha Veijola, Juha Auvinen, Jouko Miettunen, Terence M. Dovey, Marjo-Riitta Järvelin, Alexandra I. F. Blakemore

**Affiliations:** 1 Section of Investigative Medicine, Division of Diabetes, Endocrinology, and Metabolism, Faculty of Medicine, Imperial College London, London, United Kingdom; 2 Department of Life Sciences, College of Health and Life Sciences, Brunel University London, London, United Kingdom; 3 Center for Life Course Health Research, Faculty of Medicine, University of Oulu, Oulu, Finland; 4 Biocenter Oulu, University of Oulu, Oulu, Finland; 5 Unit of Primary Health Care and Medical Research Center, Oulu University Hospital, Oulu, Finland; 6 Medical Research Center Oulu, Oulu University Hospital and University of Oulu, Oulu, Finland; 7 Department of Psychiatry, Research Unit of Clinical Neuroscience, Oulu University Hospital and University of Oulu, Oulu, Finland; 8 Department of Epidemiology and Biostatistics, MRC-PHE Centre for Environment & Health, School of Public Health, Imperial College London, London, United Kingdom; Dasman Diabetes Institute, KUWAIT

## Abstract

**Background:**

Alexithymia, a difficulty in identifying and expressing emotions, has been associated with obesity and eating disorders in small-scale cross-sectional studies. Here, we assess the relationship between body mass index (BMI) and alexithymia in a large cohort of free-living Finnish adults over a 15-year period.

**Methods:**

Participants were drawn from the Northern Finnish Birth Cohort 1966 (NFBC1966). The 20-Item Toronto Alexithymia Scale (TAS-20) was used as a measure of alexithymia and was completed at the age of 31 years (31y: n = 4841), and 46 years (46y: n = 5404). BMI was recorded at both time points. Where data at both time points were available (n = 3274), the relationship between changes in BMI and TAS-20 over this time period was also investigated.

**Results:**

BMI was significantly and positively associated with TAS-20 score (p<0.0001, both at 31 years and at 46 years of ages). The association remained statistically significant after adjustment for potential confounders (sex, marital status and several socio-economic indicators). In individuals who experienced the greatest change in BMI (in either direction) over the 15-year period, there was a modest mean increase in TAS-20 score.

**Conclusions:**

Our data revealed that TAS-20 score was correlated with and co-varied with body mass status. We suggest that future clinical research should consider the role of alexithymia in obesity. Further investigation of this relationship is warranted to ensure that the needs of obese subjects with undiagnosed alexithymia are considered in the design of weight management programmes.

## Introduction

It has been previously reported that obese individuals, along with those with eating disorders, have an inability to recognise, describe and distinguish their own emotions—known as alexithymia [1, 2]. Alexithymia (a term from the Greek meaning “no words for emotions”) is a personality construct that comprises emotional and cognitive differences in ability to recognise and express one’s own emotions, with current estimates placing prevalence rates of clinical alexithymia at 10% of adult Europeans [3]. Alexithymia is characterised as a set of consistent behaviours believed to be indicative of maladaptive, and potentially dysfunctional, emotion processing [4]. It is typically screened for using psychometric measures, with the 20-item Toronto Alexithymia Scale (TAS-20) being the most common instrument [5, 6]. Larger cohort scale studies, such as that by Mattila [7] have revealed that the expected mean population TAS-20 score was 45.8. Higher TAS-20 score indicate more difficulties with identifying and describing emotions, and a threshold of TAS-20 score above 60 defines clinically-significant alexithymia. Sex differences have also been frequently reported, with males scoring significantly higher than females [8, 9].

Obesity has been reported to be associated with alexithymia [10, 11] as has anorexia nervosa [2, 12], and alexithymia is a key feature in autism [13]. However, the mechanisms underlying the association between alexithymia and obesity remain to be elucidated. Inconsistent associations between alexithymia and obesity have been reported in different populations. Small scale case-control studies in Swedish and Italian populations have indicated that the prevalence of alexithymia in obese patients is higher than in healthy individuals [10, 11] although this finding is not consistently reported across all European countries [14, 15].

Despite a wealth of data available in eating disorder patients [2], little is known about the relationship between “normal” inter-individual variation in TAS-20 score and BMI in non-clinical cohorts. Research, thus far, has concentrated on alexithymia in cross-sectional examinations of clinical populations. Moreover, investigations of the relationship between changes in BMI and TAS-20 score over a lengthy time period are notably absent. Therefore, little is known about the stability of this personality construct over an extended time period. Furthermore, understanding the relationship between maladaptive emotion processing and BMI in the general population is of importance to provide clarity on inferences drawn about clinical populations. Accordingly, we have carried out a cross-sectional and longitudinal analyses of the relationship between BMI and TAS-20 score in an adult European population to determine whether i) BMI or ii) BMI change was associated with TAS-20 score.

## Materials and methods

### Study design and sample

This study is part of the Northern Finland Birth Cohort 1966 (NFBC1966) project. NFBC1966 is a general population-based birth cohort representing 96% of live births within two provinces in Finland (Oulu and Lapland) with expected delivery date fell in between 1 January 1966 and 31 December 1966) [16]. All children were followed-up from birth until the age of 46 years at defined time points. A wide range of data were gathered using questionnaires and/or clinical examinations, but only data at 31 years (postal questionnaire data received; N = 8767, clinical examination data received; N = 6033) and 46 years (postal questionnaire data received; N = 6868, clinical examination data received; N = 5861) time points were used in this study. TAS-20 questionnaires were administered via postal inquiry at 31-year (1997) and 46-year (2012) time points. Written informed consent was obtained from all participants either via postal inquiry or clinical examination at 31-year and 46-year time points. Ethical approval for the NFBC1966 project was obtained from the Ethical Committee of the Medical Faculty of University of Oulu and Northern Ostrobothnia Hospital District.

Participants were included in this study if they had BMI and TAS-20 data available (and were not pregnant) at both 31 year time point (1997; n = 4840) and 46 year time point (2012; n = 5421). Thus, 3274 (62.9%) of the total potential population (1396 males and 1878 females) were included in the longitudinal analysis of BMI change and TAS-20.

The socio-demographic variables from the NFBC1966 cohort included in this study were marital status, education, employment status and household income level. Marital status was classified as: i) married, ii) cohabiting, iii) single, iv) divorced, or v) widowed. Educational background was divided into four classes: from i) no education or unfinished basic education, ii) completed 9-year basic education with or without vocational training or vocational school or post-secondary school, iii) completed 9-year basic education or matriculation examination with or without polytechnic education to iv) completed 9-year basic education or matriculation examination with university degree. The employment status was categorised into three classes: i) employed, ii) unemployed, and iii) “others” (students, retired, and subjects on paternity or maternity leave). The reported family gross income in the previous year was divided into quartiles and used as income level.

### TAS-20 score

The TAS-20 questionnaire has a three-factor structure, consisting of Difficulty Identifying Feelings (DIF), Difficulty Describing Feelings (DDF), and Externally Oriented Thinking (EOT). TAS-20 scores are normally distributed in the NFBC1966 population at both 31-year and 46-year time points. Alexithymia was determined as TAS-20 score ≥61[6]. Change in TAS-20 was calculated as ΔTAS-20 = TAS-20_46y_ –TAS-20_31y._ The scale showed good internal consistency in Finnish translation for TAS-20 score (Cronbach’s alpha = 0.83), DIF (Cronbach’s alpha = 0.81), DDF (Cronbach’s alpha = 0.77) and EOT (Cronbach’s alpha = 0.66) [17].

### Body mass index

**Fig 1:**
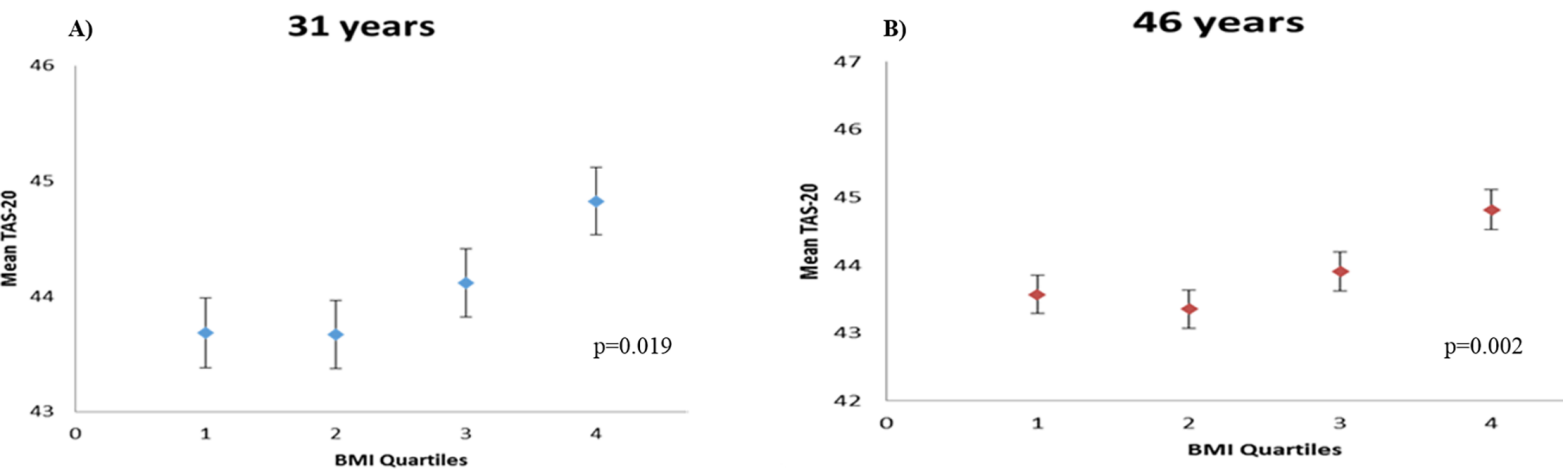
Cross-sectional analysis of TAS-20 scores and BMI at A) 31-year and B) 46-year time points in the NFBC1966. BMI was subdivided into quartiles at each time point measured; Q1_31y_: 15.32–21.75 kg/m^2^, Q2_31y_: 21.76–23.86 kg/m^2^, Q3_31y_: 23.87–26.50 kg/m^2^, Q4_31y_: 26.51–54.32 kg/m^2^, Q1_46y_: 16.06–23.57 kg/m^2^, Q2_46y_: 23.58–26.15 kg/m^2^, Q3_46y_: 26.16–29.41 kg/m^2^ and Q4_46y_: 29.42–73.81 kg/m^2^. Data presented are estimated marginal means (±SEM) derived from the model, adjusted for sex and socio-economic status (marital status, education level, employment status and annual income).

**Fig 2:**
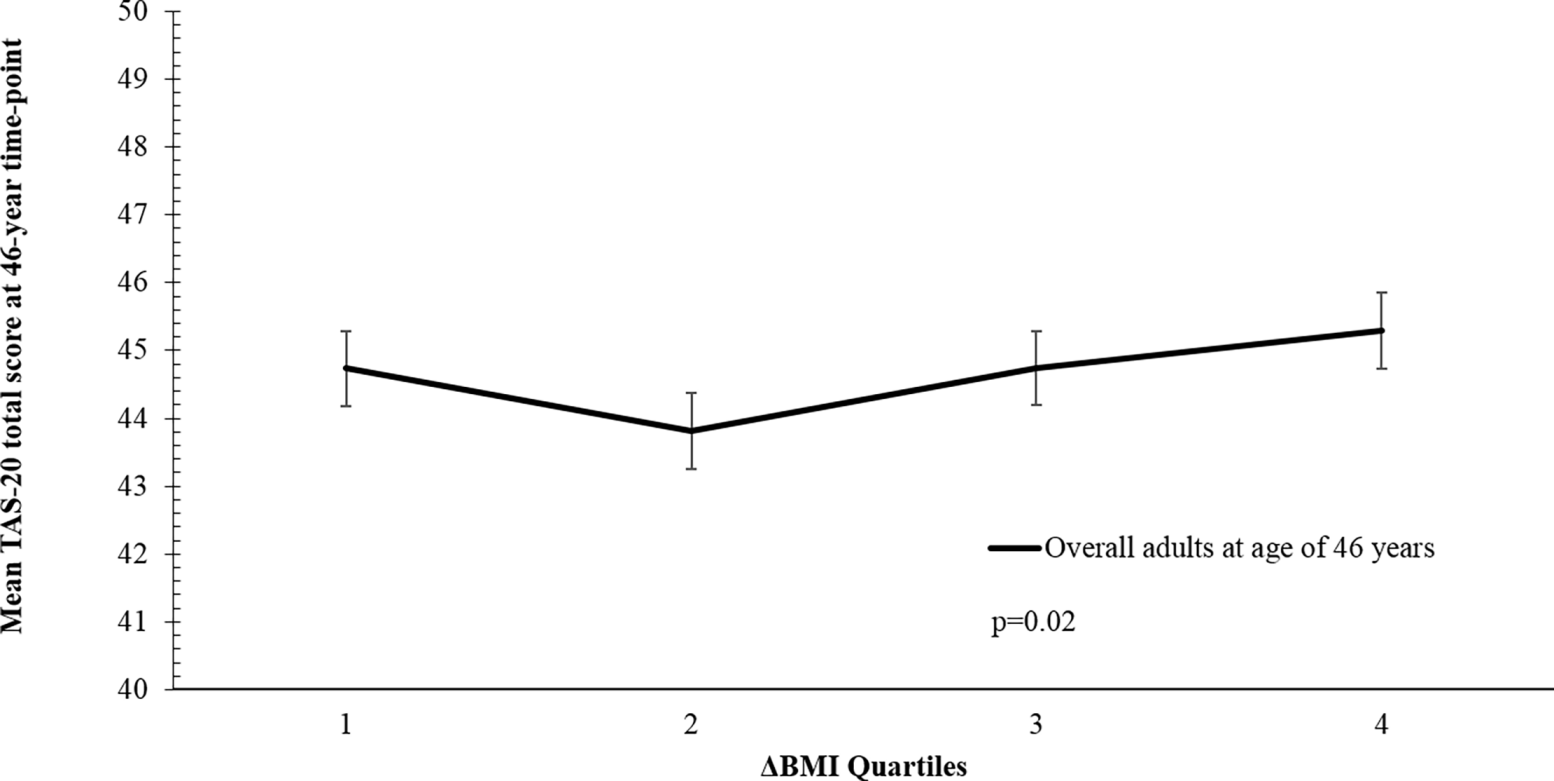
Longitudinal analysis of TAS-20 scores at 46-year time point and change of BMI quartiles in the NFBC1966. ΔBMI was subdivided into quartiles with cut-off points for Q1: -19.69–0.57 kg/m^2^, Q2: 0.58–2.06 kg/m^2^, Q3: 2.07–3.79 kg/m^2^, Q4: 3.80–23.24 kg/m^2^. Data presented are estimated marginal means (±SEM) derived from the model, adjusted for covariates (sex, marital status, education level and annual income).

BMI, based on clinical examination, was calculated as weight (in kilograms) divided by height (in metres) squared (kg/m^2^) and BMI groups were according to the WHO International Classification system [18]. BMI was also subdivided into quartiles at each time point; Q1_31y_: 15.32–21.75 kg/m^2^, Q2_31y_: 21.76–23.86 kg/m^2^, Q3_31y_: 23.87–26.50 kg/m^2^, Q4_31y_: 26.51–54.32 kg/m^2^, Q1_46y_: 16.06–23.57 kg/m^2^, Q2_46y_: 23.58–26.15 kg/m^2^, Q3_46y_: 26.16–29.41 kg/m^2^ and Q4_46y_: 29.42–73.81 kg/m^2^ (Fig 1). BMI change was calculated as ΔBMI = BMI_46y_ –BMI_31y_ and subdivided into quartiles (Fig 2) with cut-off points for Q1: -19.69–0.57 kg/m^2^, Q2: 0.58–2.06 kg/m^2^, Q3: 2.07–3.79 kg/m^2^, Q4: 3.80–23.24 kg/m^2^.

### Statistical analyses

TAS-20 and BMI data are presented as means (±SD). Analysis of covariance (ANCOVA) and Pearson correlation (r) were undertaken to assess the relationship between BMI and TAS-20 score. Sex, marital status and several other socio-economic indicators were adjusted for as potential confounders. All statistical tests were two-tailed with an alpha set to 0.05 level. Statistical analyses were conducted with SPSS v.20.0 (Armonk, NY: IBM Corp).

## Results

From the whole NFBC1966 cohort, 4841 and 5404 participants responded to both TAS-20 and socio-demographic questionnaires (Tables 1 and 2), of which 6.9% and 6.5% passed the threshold for alexithymia (assessed as a binary trait, clinical cut-off TAS-20≥ 61), at the ages of 31 years and 46 years respectively. Those above the threshold for alexithymia at 31 years showed an average decrease of 8.48 points at 46 years (p<0.0001). The mean values for BMI, at age 31 years and 46 years, were 24.29 (±4.25) kg/m^2^ and 26.68 (±4.94) kg/m^2^, respectively. Individuals with the smallest change in BMI (+/- 5% kg/m^2^) (n = 888) had, on average, stable mean TAS-20 score over time (43.6±10.1 and 43.7±9.8, at 31 and 46 years respectively). In the longitudinal set (n = 3274), there was a difference in the prevalence of alexithymia between 31 years (n = 199, 6.1%) and 46 years (n = 206, 6.3%) (p<0.0001) (S1 Table). However, only 2.2% of the total population (n = 71) remained in the clinically-relevant alexithymic category at both time points.

**Table 1:** Demographic table of NFBC1966 participants at the 31-year time point with available data.

Subject Characteristics	31-year N = 4841	Non-Alexithymia N = 4507	Alexithymia N = 334	*P-value
**Male/Female (%)**	2277/2564 (47.0/53.0)	2069/2438 (45.9/54.1)	208/126 (62.3/37.7)	**<0.0001**
**BMI, kg/m**^**2**^ **(**±**SD)**	24.6 (±4.3)	24.5 (±4.2)	25.5 (±5.1)	**<0.0001**
**TAS-20**	44.0 (±10.2)	42.7 (±8.8)	65.5 (±4.1)	**<0.0001**
DIF	13.4 (±4.7)	12.8 (±4.1)	22.8 (±3.9)	**<0.0001**
DDF	10.8 (±3.9)	10.4 (±3.5)	17.7 (±2.3)	**<0.0001**
EOT	19.7 (±4.7)	19.5 (±4.5)	25.1 (±3.5)	**<0.0001**
**Marital status (%)**				**<0.0001**
Married	2265 (47.1)	2152 (48.1)	113 (34.1)	
Cohabiting	1179 (24.5)	1116 (24.9)	63 (19.0)	
Single	1166 (24.3)	1019 (22.8)	147 (44.4)	
Divorced	193 (4.0)	185 (4.1)	8 (2.4)	
Widowed	3 (0.1)	3 (0.1)	0	
**Education (%)**				**<0.0001**
1—low	22 (0.3)	12 (0.2)	10 (3.0)	
2	2526 (51.1)	2292 (48.4)	234 (70.7)	
3	1760 (37.6)	1686 (39.2)	74 (22.4)	
4—high	511 (11.1)	498 (12.2)	13 (3.9)	
**Income (%)**				**<0.0001**
1—low	1120 (25.7)	1008 (24.7)	112 (40.0)	
2	1186 (27.2)	1120 (27.5)	66 (23.7)	
3	1000 (23.0)	945 (23.2)	55 (19.7)	
4—high	1049 (24.1)	1003 (24.6)	46 (16.5)	
**Occupation (%)**				**<0.0001**
Employed	3327 (69.3)	3128 (70.0)	199 (60.0)	
Unemployed	816 (17.0)	733 (16.4)	83 (25.0)	
Others	655 (13.7)	605 (13.5)	50 (15.0)	
**BMI status (%)**				**0.004**
Underweight	117 (2.4)	106 (2.4)	11 (3.3)	
Normal	2824 (58.3)	2657 (59.0)	167 (50.9)	
Overweight	1469 (30.3)	1356 (30.1)	113 (33.8)	
Obesity	431 (8.9)	388 (8.6)	43 (12.9)	

Data is presented as mean (±SD) for continuous (TAS-20 and BMI) variables and as a percentage for categorical variable. TAS-20 is the total score of the 20-item Toronto Alexithymia Scale questionnaire. Alexithymia status was defined by a total TAS-20 ≥61. DIF: difficulty identifying feelings. DDF: difficulty describing feelings. EOT: externally oriented thinking. BMI ranges were classified according to the WHO International Classification system [18] that defines the following categories: underweight (UW) (<18.5 kg/m^2^), normal weight (18.5–24.9 kg/m^2^), overweight (OW) (25–29.9 kg/m^2^) and obesity (OB) (≥30 kg/m^2^).

*Comparisons between alexithymia groups used 2-sided independent t-test for continuous variables and χ^2^ test between categorical variables.

**Table 2:** Demographic table of NFBC1966 participants at the 46-year time point with available data.

Subject Characteristics	46-year N = 5404	Non-Alexithymia	Alexithymia	*P-value
		N = 5053	N = 351	
**Male/Female (%)**	2382/3022 (44.0/56.0)	2172/2881 (43.0/57.0)	210/141 (60.0/40.0)	**<0.0001**
**BMI, kg/m**^**2**^ **(**±**SD)**	26.8 (±4.9)	26.7 (±4.8)	28.2 (±6.0)	**<0.0001**
**TAS-20**	44.1 (±10.1)	42.6 (±8.6)	65.5 (±4.4)	**<0.0001**
DIF	13.3 (±4.7)	12.7 (±4.0)	22.7 (±3.7)	**<0.0001**
DDF	10.8 (±3.7)	10.3 (±3.3)	17.9 (±2.2)	**<0.0001**
EOT	20.1 (±4.4)	19.7 (±4.3)	24.9 (±3.1)	**<0.0001**
**Marital status (%)**				**<0.0001**
Married	3141 (60.0)	2959 (60.5)	182 (53.2)	
Cohabiting	972 (18.6)	911 (18.6)	61 (17.8)	
Single	594 (11.4)	525 (10.7)	69 (20.2)	
Divorced	506 (9.7)	476 (9.7)	30 (8.8)	
Widowed	20 (0.4)	20 (0.4)	0	
**Education (%)**				**<0.0001**
1—low	80 (1.5)	69 (1.4)	10 (3.2)	
2	1703 (32.5)	1523 (31.1)	180 (52.5)	
3	1150 (21.9)	1086 (22.1)	64 (18.7)	
4—high	2313 (44.1)	2225 (45.4)	88 (25.6)	
**Income (%)**				**<0.0001**
1—low	1066 (22.4)	971 (21.8)	95 (31.8)	
2	1227 (25.8)	1144 (25.6)	83 (27.8)	
3	1084 (22.8)	1008 (22.6)	76 (25.4)	
4—high	1383 (29.1)	1338 (30.0)	45 (15.1)	
**Occupation (%)**				**<0.0001**
Employed	4619 (89.0)	4365 (89.9)	254 (75.8)	
Unemployed	292 (5.6)	249 (5.1)	43 (12.8)	
Others	279 (5.4)	241 (5.0)	38 (11.3)	
**BMI status (%)**				**<0.0001**
Underweight	35 (6.5)	32 (0.6)	3 (0.9)	
Normal	2094 (38.7)	1992 (39.4)	102 (29.1)	
Overweight	2144 (39.7)	2009 (39.8)	135 (38.5)	
Obese	1131 (20.9)	1020 (20.2)	111 (31.6)	

Data is presented as mean (±SD) for continuous (TAS-20 and BMI) variables and as a percentage for categorical variable. TAS-20 is the total score of the 20-item Toronto Alexithymia Scale questionnaire. Alexithymia status was defined by a total TAS-20 ≥61. DIF: difficulty identifying feelings. DDF: difficulty describing feelings. EOT: externally oriented thinking. BMI ranges were classified according to the WHO International Classification system [18] that defines the following categories: underweight (UW) (<18.5 kg/m^2^), normal weight (18.5–24.9 kg/m^2^), overweight (OW) (25–29.9 kg/m^2^) and obesity (OB) (≥30 kg/m^2^).

*Comparisons between alexithymia groups used 2-sided independent t-test for continuous variables and χ^2^ test between categorical variables.

As expected, males scored higher on the TAS-20 scale compared to females at both time points (31y: p<0.001, 46y: p<0.001). Participants with clinically-relevant alexithymia had higher BMI at both time points (31y: p = 0.001, 46y: p<0.001). There was also an association between BMI and TAS-20 score itself across the whole population. Fig 1 shows the relationship between BMI at 31 and 46 years’ time points and TAS-20 scores. TAS-20 scores associated with both BMI quartiles (31y: p<0.0001, 46y: p<0.0001) and WHO-defined BMI groups (31y: p<0.0001, 46y: p<0.0001) at each time point. When adjusted for sex, marital status, education, employment status and household income level at 31 years, the statistical differences in mean TAS-20 score between BMI quartiles (31y: p = 0.019, 46y: p = 0.002) and between BMI groups (31y: p = 0.019, 46y: p = 0.003) remained consistent. Correlation analysis for TAS-20 score at 31- and 46-year time points showed consistent positive, but weak, associations with BMI (31y: r_(4841)_ = 0.10, p<0.0001, 46y: r_(5404)_ = 0.11, p<0.0001).

Significant differences in TAS-20 score between BMI quartiles at 31 years were specifically due to the DIF and EOT subscales (DIF_31y_: p = 0.005, EOT_31y_: p<0.0001) of the TAS-20, which indicated that higher BMI individuals reported, on average, more difficulty in identifying feelings, and more externally-oriented thinking. Significant differences in mean TAS-20 score were observed between participants who had normal BMI (18.5–24.9 kg/m^2^) and those who were obese (BMI> 30 kg/m^2^) at the 31-year time point (p<0.0001).

Longitudinal changes in BMI (ΔBMI) and TAS-20 score over the 15-year interval between the two time points showed a U-shaped relationship between quartiles of ΔBMI and mean TAS-20 score at the 46-year time point (see Fig 2). Participants with the greatest ΔBMI (in a positive or negative direction) over the 15-year test period had higher mean TAS-20 score at age of 46 years (p = 0.003). A sex difference was observed in ΔBMI (p<0.0001). Over the 15-year study period, 4.3% of females (n = 141) remained in the obese category, as shown in Table 3, compared to 37.6% of males (n = 85). Females and males, on average, gained 2.66 (±3.25) kg/m^2^ and 2.02 (±2.56) kg/m^2^ respectively and sex difference was seen in overall BMI comparison between both time points (31y: p<0.001, 46y: p = 0.002) (S1 Table). The same pattern of sex differences was manifested in EOT subscale for all of three BMI transitions groups at both time points (all p<0.0001).

**Table 3:** Transition between body mass index groups in NFBC1966 participants between 31-year and 46-year time points.

**Remained in** **obesity status**	**31 years**				**46 years**				P-value*
	**Males**	**Females**	^**¥**^**P-value**	**Overall**	**Males**	**Females**	^**¥**^**P-value**	**Overall**	
**N (%)**	85 (37.6)	141 (62.4)		226	85 (37.6)	141 (62.4)		226	
**TAS-20**	47.5 (±11.4)	44.5 (±10.4)	**0.041**	45.6 (±10.9)	49.2(±11.0)	44.4(±10.5)	**0.001**	46.2 (±10.9)	0.213
**DIF**	13.6 (±5.5)	14.5 (±5.2)	0.217	14.2 (±5.3)	14.8 (±5.8)	14.5 (±5.1)	0.643	14.6 (±5.3)	0.131
**DDF**	12.0 (±4.2)	10.8 (±4.1)	**0.041**	11.2 (±4.2)	12.2 (±4.0)	10.6 (±3.5)	**0.001**	11.2 (±3.8)	0.971
**EOT**	21.9 (±4.8)	19.2 (±4.5)	**<0.0001**	20.2 (±4.8)	22.2 (±3.9)	19.4 (±4.3)	**<0.0001**	20.5 (±4.4)	0.327
**Overweight/ obesity → normal/ underweight**	**31 years**				**46 years**				P-value*
	**Males**	**Females**	^**¥**^**P-value**	**Overall**	**Males**	**Females**	^**¥**^**P-value**	**Overall**	
**N (%)**	38 (50.7)	37 (49.3)		75	38 (50.7)	37 (49.3)		75	
**TAS-20**	47.4 (±9.7)	40.6 (±7.7)	**0.001**	44.0 (±9.4)	47.3 (±8.7)	43.0 (±11.1	0.065	45.2 (±10.2)	0.190
**DIF**	13.6 (±4.9)	13.1 (±3.8)	0.604	13.3 (±4.3)	14.0 (±4.7)	13.7 (±4.7)	0.748	13.9 (±4.7)	0.271
**DDF**	11.4 (±4.1)	9.7 (±4.2)	0.067	10.6 (±4.2)	11.6 (±3.8)	11.3 (±4.2)	0.805	11.4 (±4.0)	**0.040**
**EOT**	22.3 (±3.5)	17.8 (±3.3)	**<0.0001**	20.1 (±4.1)	21.8 (±3.7)	18.0 (±4.3)	**<0.0001**	19.9 (±4.4)	0.661
**Normal/ underweight → overweight/ obesity**	**31 years**				**46 years**				P-value*
	**Males**	**Females**	^**¥**^**P-value**	**Overall**	**Males**	**Females**	^**¥**^**P-value**	**Overall**	
**N (%)**	342 (39.4)	526 (60.6)		868	342 (39.4)	526 (60.6)		868	
**TAS-20**	46.4 (±9.7)	42.3 (±10.2)	**<0.0001**	43.9 (±10.2)	46.7 (±9.4)	42.4 (±9.6)	**<0.0001**	44.1 (±9.8)	0.560
**DIF**	13.2 (±4.4)	13.8 (±4.8)	0.079	13.6 (±4.7)	13.4 (±4.5)	13.4 (±4.6)	0.876	13.4 (±4.6)	0.284
**DDF**	11.8 (±4.0)	10.1 (±3.7)	**<0.0001**	10.7 (±3.9)	11.7 (±3.7)	10.2 (±3.7)	**<0.0001**	10.8 (±3.8)	0.751
**EOT**	21.4 (±4.6)	18.4 (±4.4)	**<0.0001**	19.6 (±4.7)	21.5 (±4.0)	18.8 (±4.2)	**<0.0001**	20.0 (±4.3)	**0.029**

Data is presented as mean (±SD) for continuous variables. TAS-20 is the total score of the 20-item Toronto Alexithymia Scale questionnaire. Alexithymia status was defined by a total TAS-20 ≥61. DIF: difficulty identifying feelings. DDF: difficulty describing feelings. EOT: externally oriented thinking.

^¥^Comparisons between sex at each time point used 2-sided independent t-test for continuous variables and a χ^2^ test between categorical variables.

*Overall comparison between both time points used 2-sided paired t-test and χ^2^ tests.

## Discussion

Here, we report a positive association between BMI and TAS-20 (as an overall score and subscales) in a large population-based cohort of European adults examined at both 31 years and 46 years of age. In addition, exploration of the changes in BMI and TAS-20 over a period of 15 years showed that the relationship between BMI change and TAS-20 score is significant at the 46-year time point. In addition, participants who had the greatest decrease or increase in BMI had higher mean TAS-20 scores (indicating lower emotional processing function), and, conversely, those with stable TAS-20 scores also had more stable BMIs. Mean differences, corroborating the correlation, indicated that the TAS-20 score increased, on average, by roughly one point for every quartile increase in BMI. There was a high variability of TAS-20 scores from the same individuals over the 15 year time period (data not shown). Only 71 participants met the criteria for alexithymia at both time points measured, indicating that the TAS-20 scale was not measuring a stable psychological trait as previously thought [19, 20]. We observed that TAS-20 scores altered over time and co-varied with concomitant changes in weight.

Significant sex differences were observed in TAS-20 scores, as reported in previous alexithymia studies in the Finnish population [9, 21], the proportion of males (54% of the total population above the threshold) was marginally higher in alexithymia cases. There have been a few neurobiological studies investigating sex difference in emotions [22–24]. Lumley and Sielky [25] suggested alexithymia has a different aetiology for males and females. They proposed that alexithymia characteristics are based on biological factors (hemispheric functioning) for males, however in females they suggest it is more likely to be developed as a consequence of emotional trauma [25].

Alexithymia has been associated with obesity and eating disorders [1, 12, 26, 27] but there have been inconsistent results in different population groups. de Zwaan [14] and Golab [15] observed that alexithymia measures did not differ between obese Polish and Austrian subjects with or without binge eating disorder respectively. However, previous small-scale case-control studies indicated that the prevalence of alexithymia in obese patients is higher than in healthy individuals [10, 11]. Nowakowski (2013)[2] and Westwood (2017)[28] suggested DDF and DIF subscales as trans-diagnostic criteria for emotional dysregulation across the eating disorder spectrum (anorexia nervosa, bulimia nervosa and binge-eating disorder). However, for patients with anorexia, scores of TAS-20 and its subscales (DDF, DIF and EOT) were significantly higher than those of normal-weight controls [28]. Other researchers reported no significant difference in TAS-20 scores between recovered anorexia patients and healthy controls [29]. This suggests that starvation or malnutrition in the acute phase of anorexia may affect alexithymia scores [28], reflecting deficits in cognitive function and emotional regulation, based on the generally accepted James Gross' models of emotion regulation [30, 31].

A possible contribution to the increasing rate of adult obesity is poor emotion handling, which may negatively influence healthy behaviour and lifestyle. Current findings suggest that consideration of alexithymia diagnosis may help in design of treatment strategies for morbidly obese patients [32], since alexithymic individuals have been shown to have poorer nutritional intake and decreased immune functioning [33]. Previous studies in the Northern Finland Birth Cohort 1966 have explored the connections among obesity, depression, psychosocial function and working ability [34–36]. We found the same weight trajectories as in Nevanperä [35] reporting that the obesity prevalence rate doubled over a 15-year period.

One potential limitation is that the relationship that we observed might have been confounded by lifestyle, physical and/or psychiatric conditions including smoking, drug use, co-morbid conditions, locus of control and other factors. Since depression has been separately associated with obesity and with alexithymia [37, 38], future studies will be needed to investigate TAS-20 score in subjects with and without depression, and specifically how this interacts with BMI status and weight change. In addition, the observation of changed individual TAS-20 score (previously considered to reflect a stable personality trait) over the test period, raises the possibility of designing specific strategies to improve emotion-handling capacity, for inclusion in interventions for obesity or to promote healthy regulation of eating behaviours.

Future studies regarding the association between changes in BMI/weight and emotion-processing are needed, for the direction of causality to be fully determined. To date, there has been no information in the literature exploring the phenotypic and genetic profile of alexithymia and BMI simultaneously over the life course in general populations. Mendelian randomisation and other causal studies could be conducted to determine the directions of causality between alexithymia, depression and obesity. We also speculate that the development of obesity in some cases may be associated with undiagnosed alexithymia. Deficits in emotion-processing should, therefore, be considered in the design of weight-management programmes.

## Supporting information

S1 Table Descriptive statistics by sex of the longitudinal set at both 31-year and 46-year time points (n = 3274). (DOCX)
