# Supplementary material for: Relationship between BMI and emotion-handling capacity in an adult Finnish population: The Northern Finland Birth Cohort 1966
Source: PLoS One. 2018 Sep 26;13(9):e0203660. doi: 10.1371/journal.pone.0203660 (PMC6157858; doi:10.1371/journal.pone.0203660)
Supplement: S1 Table — (DOCX) [file pone.0203660.s001.docx]

S1 Table. Descriptive statistics by sex of the longitudinal set at both 31-year and 46-year time points (n=3274).

|  | | | | | | | | | | | |
| --- | --- | --- | --- | --- | --- | --- | --- | --- | --- | --- | --- |
|  | **31 years** | | | |  | **46 years** | | | |  | ***P-value** |
|  | **Males** | **Females** | **^¥^P-value** | **Overall** |  | **Males** | **Females** | **^¥^P-value** | **Overall** |  |  |
| **N (%)** | 1396 (42.6) | 1878 (57.4) |  | 3274 |  | 1396 (42.6) | 1878 (57.4) |  | 3274 |  |  |
| **BMI** | 25.1 (±3.4) | 24.0 (±4.6) | **<0.0001** | 24.5 (±4.1) |  | 27.1 (±4.1) | 26.7 (±5.5) | **0.002** | 26.8 (±4.9) |  | **<0.0001** |
| **TAS-20** | 46.3 (±9.7) | 42.2 (±10.0) | **<0.0001** | 43.9 (±10.1) |  | 46.8 (±9.6) | 42.2 (±9.7) | **<0.0001** | 44.1 (±9.9) |  | 0.205 |
| **DIF** | 13.1 (±4.5) | 13.7 (±4.7) | **<0.0001** | 13.4 (±4.6) |  | 13.3 (±4.6) | 13.2 (±4.6) | 0.349 | 13.2 (±4.6) |  | **0.015** |
| **DDF** | 11.7 (±3.9) | 10.2 (±3.7) | **<0.0001** | 10.8 (±3.9) |  | 11.7 (±3.7) | 10.1 (±3.6) | **<0.0001** | 10.8 (±3.7) |  | 0.372 |
| **EOT** | 21.5 (±4.4) | 18.4 (±4.4) | **<0.0001** | 19.7 (±4.6) |  | 21.8 (±3.9) | 18.9 (±4.2) | **<0.0001** | 20.1 (±4.3) |  | **0.005** |
| **Alexithymia cases (%)** | 110 (7.9) | 89 (4.7) | **<0.0001** | 199 (6.1) |  | 114 (8.2) | 92 (4.9) | **<0.0001** | 206 (6.3) |  | **<0.0001** |

Data is presented as mean (±SD) for continuous variables and as a percentage for categorical variables. TAS-20 is the total score of the 20-itemToronto Alexithymia Scale questionnaire. Alexithymia case was defined by TAS-20 ≥61. DIF: difficulty identifying feelings. DDF: difficulty describing feelings. EOT: externally oriented thinking.

**^¥^**Comparisons between sex at each time point used 2-sided independent t-test for continuous variables and a χ^2^ test between categorical variables.

*Overall comparison between both time points used 2-sided paired t-test for continuous variables and χ^2^ test between categorical variables.
